# Massively parallel sequencing of cell-free DNA in plasma for detecting gynaecological tumour-associated copy number alteration

**DOI:** 10.1038/s41598-018-29381-y

**Published:** 2018-07-25

**Authors:** Makoto Nakabayashi, Akihiro Kawashima, Rika Yasuhara, Yosuke Hayakawa, Shingo Miyamoto, Chiaki Iizuka, Akihiko Sekizawa

**Affiliations:** 1 Department of Obstetrics and Gynecology, Showa University School of Medicine, 1-5-8 Hatanodai, Shinagawa-ku, Tokyo, 142-8666 Japan; 2 Division of Pathology, Department of Oral Diagnostic Sciences, Showa University School of Dentistry, 1-5-8 Hatanodai, Shinagawa, Tokyo, 142-8666 Japan; 3 Information System Department GeneTech, Inc. 2-6-7 Kazusa-Kamatari, Kisarazu, Chiba, 292-0818 Japan

## Abstract

The discovery of circulating tumour DNA molecules created a paradigm shift in tumour biomarkers as predictors of recurrence. Non-invasive prenatal testing (NIPT) to detect circulating cell-free foetal DNA in maternal plasma is increasingly recognised as a valuable substitute to perceive foetal copy number variation (CNV). This study aimed to determine whether the copy number detection in plasma samples using NIPT platform could be used as a prognostic biomarker in patients with gynaecological cancer. We conducted a prospective study using samples containing preoperative plasma from 100 women with gynaecological cancers. Samples were randomly rearranged and blindly sequenced using a low-coverage whole-genome sequencing plasma DNA, NIPT platform. The NIPT pipeline identified copy number alterations (CNAs) were counted in plasma as a gain or loss if they exceeded 10 Mb from the expected diploid coverage. Progression-free survival (PFS) and overall survival (OS) were analysed according to the presence of CNA in plasma using Kaplan–Meier analyses. The NIPT pipeline detected 19/100 cases of all gynaecological cancers, including 6/36 ovarian cancers, 3/11 cervical cancers, and 10/53 endometrial cancers. Patients with CNA in plasma had a significantly poorer prognosis in all stages concerning PFS and OS. Therefore, low-coverage sequencing NIPT platform could serve as a predictive marker of patient outcome.

## Introduction

In recent years, cell-free DNA has been broadly studied using circulating tumour DNA (ctDNA) as a liquid biopsy, including the detection of minimal residue, early detection of resistance to therapy, early detection of disease and assessment of molecular heterogeneity. Occult maternal malignancies can be detected via non-invasive prenatal testing (NIPT) using massively parallel sequencing (MPS) of cell-free DNA from the maternal plasma for prenatal screening of common foetal autosomal aneuploidies and trisomies 21, 18 and 13. In many cases, the cell-free DNA in the plasma of pregnant women is a mixture of placental and maternal DNA. Follow-up studies have demonstrated that some cell-free DNA events are discordant with the direct foetal karyotype and may detect asymptomatic neoplasms in the mothers^1^. One such example involves a patient who was diagnosed with metastatic small cell carcinoma of the vagina that was suggested to account for aneuploidies of chromosome 18 and 13 identified using NIPT^2^. In other reports, cell-free DNA discordances were determined using MPS for NIPT, and two patients with Hodgkin’s Disease^3,4^ were identified. In a recent study, use of a clinical NIPT platform detected early-stage ovarian cancer^5^. As potential biological explanations for cell-free DNA discordance include confined malignancy, this suggests that genomic profiling by the NIPT platform, which is broadly used for testing foetal aneuploidies, may also represent a practical approach for clinical neoplasm management.

Several studies have revealed the presence of tumour-derived DNA in the plasma of cancer patients^6–8^. Cell-free DNA released from apoptotic cells is shortened to 185–200 bp-fragments. DNA fragments are released into the bloodstream from dying cells during cell turnover or from apoptotic and necrotic cells^9^. Under normal physiological circumstances, apoptotic and necrotic cells are cleared by infiltrating phagocytes, and cell-free DNA levels are relatively low. In solid tumours, cell-free DNA is also released via necrosis, autophagy, apoptosis and other physiological events induced by micro-environmental stress and treatment pressure^10^. This phenomenon suggests that ctDNA may be more likely to originate from genomic regions with an increased euchromatic DNA structure resulting in observed differential fragment length distribution in coverage relative to somatic cell-free DNA. Recent improvements in the analysis of blood samples for circulating tumour cells or ctDNA has provided rapid, cost-effective and non-invasive liquid biopsy surrogates, which provide valuable complementary information on therapeutic targets and drug resistance mechanisms in cancer patients^11,12^. Tumour heterogeneity introduces significant challenges in designing effective treatment strategies^13^. CNV is amplified or deleted in regions of the genome that are recognised as a primary source of average human genome viability and contribute significantly to phenotype variation. One crucial feature arising from previous studies is the observation that tumour DNA carries genomic alterations corresponding to CNA^14^. CNA plays a significant role in carcinogenesis in many cancers, such as ovarian cancer^15^, hepatocellular carcinoma^16^, and colorectal carcinoma^17^. Several studies have verified that somatic CNVs in ctDNA match those present in the primary tumour^18^.

Genome-wide detection of CNA can be characterised in ctDNA, acting as tumour biomarkers with excellent sensitivity and specificity^19,20^. These methods require deep sequencing that significantly increases the cost and difficulty to use in clinical practice. Chromosomal instability analysis in cell-free DNA by low-coverage whole-genome sequencing was used for the primary diagnosis of ovarian cancer^21^. In prenatal testing, several studies have demonstrated the possibility of using whole-genome sequencing-based NIPT to detect fetal CNV^22,23^. Recently, several studies using MPS have also reported that personalised analysis of rearranged ends was developed to detect unselected genetic events that span across the whole genome in cancer patients^24,25^. These findings demonstrate the performance of cancer genome scanning through MPS of plasma DNA.

Several prototype studies also evaluated the low-coverage sequencing method using MPS for the detection of foetal CNVs. Recently, detection of CNA using MPS was reviewed^26^. The critical advantage of MPS technologies is the reduced cost and time required to sequence a sample. This method allows for more examples to be investigated than previously possible, and an increase in available information and statistical power has resulted in the identification of many new genes thought to be involved in cancer biology. We hypothesised that CNA in plasma derived from tumours would be detected in patients with gynaecological cancer before primary surgery and would predict prognosis. This study aimed to determine whether the use of an NIPT platform for CNA in plasma from patients with gynaecological cancer could serve as a predictive marker of patient outcome.

## Results

### Assessment of SeqFF

**Figure 1:**
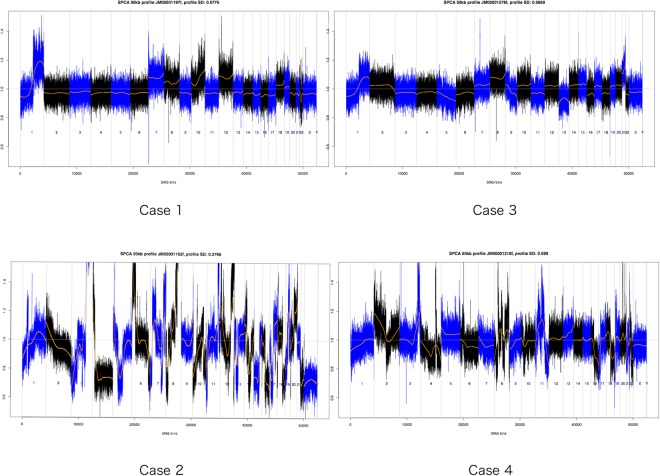
Whole genome view of copy number gains and losses in plasma samples from women positive for trisomy 13, 18 and 21 using Genetech NIPT. Smoothed normalised coverage (in orange) is plotted along Z-score (Y-axis) and the genomic coordinates (x-axis), sorted by chromosome number and genomic location within the chromosomes. Case 1: diagnosed with a stage III ovarian cancer, high-grade serous carcinoma, Case 2: a stage III ovarian cancer, high-grade serous carcinoma, Case 3: a stage I ovarian cancer, Dysgerminoma Stage I, Case 4: a stage IV corpus cancer, serous adenocarcinoma.

We enrolled 100 patients with gynaecological cancer in the study and analysed plasma samples from those with ovarian cancer (n = 36), cervical cancer (n = 11) and endometrial cancer (n = 53). We detected CNA in 1/21 early-stage ovarian cancers, 5/15 advanced stage ovarian cancers, 3/11 early-stage cervical cancer cases, 5/41 early stage endometrial cancer cases and 5/12 advanced stage endometrial cancer cases using the NIPT platform (Table 1). The Genetech NIPT platform analysis indicated that five patients were positive for at least one aneuploidy involving chromosomes 13, 18 and 21 (Fig. 1). Cases with trisomies detected by NIPT could be explained by total or sizeable partial copy number gains on the test chromosomes and chromosomes 13, 18 and 21. We found that samples from patients with advanced stages (stages III–IV) had a higher rate of CNA detection than those with early stages (stage I–II) (37.0% for stages III–IV patients versus 12.3% for stages I–II; p = 0.009). Moreover, the rate of CNA detection was higher in patients with advanced stage endometrial cancer than in those with early-stage endometrial cancer (41.6% for stages III–IV versus 12.2% for stages I–II; p = 0.035). There was no difference in the CNA range in plasma among all cancer types. These observations highlight the benefit of analysing whole-genome sequencing to increase the possibility of detecting CNA in plasma in advanced tumours.

**Table 1:** Each gynaecological cancer type and clinical stage of total samples in this sequencing analysis which calls copy number alteration using Genetech NIPT pipeline, n = 100.

FIGO Stage	Patients (n)	Trisomy 21 (n)	Trisomy 18 (n)	Trisomy 13 (n)	Detected patients with plasma CNA (n)	Plasma CNA range in the patients with detected CAN (Median, range)	Rate of patients with plasma CAN (%)
**Ovarian cancer**							
I–II	21	1	0	0	1	2448	4.8%
III–IV	15	0	1	1	5	298 (31–1767)	33.3%
**Cervical cancer**							
I–II	11	0	0	0	3	89 (21–1077)	27.3%
III–IV	N/A	N/A	N/A	N/A	N/A	N/A	N/A
**Endometrial cancer**							
I–II	41	0	0	0	5	248 (93–1492)	19.5%
III–IV	12	0	0	1	5	102 (62–834)	41.7%

Abbreviations are follow: CNA, copy number alteration, NA, not available.

### Focal alterations in gynaecological cancer genes

**Figure 2:**
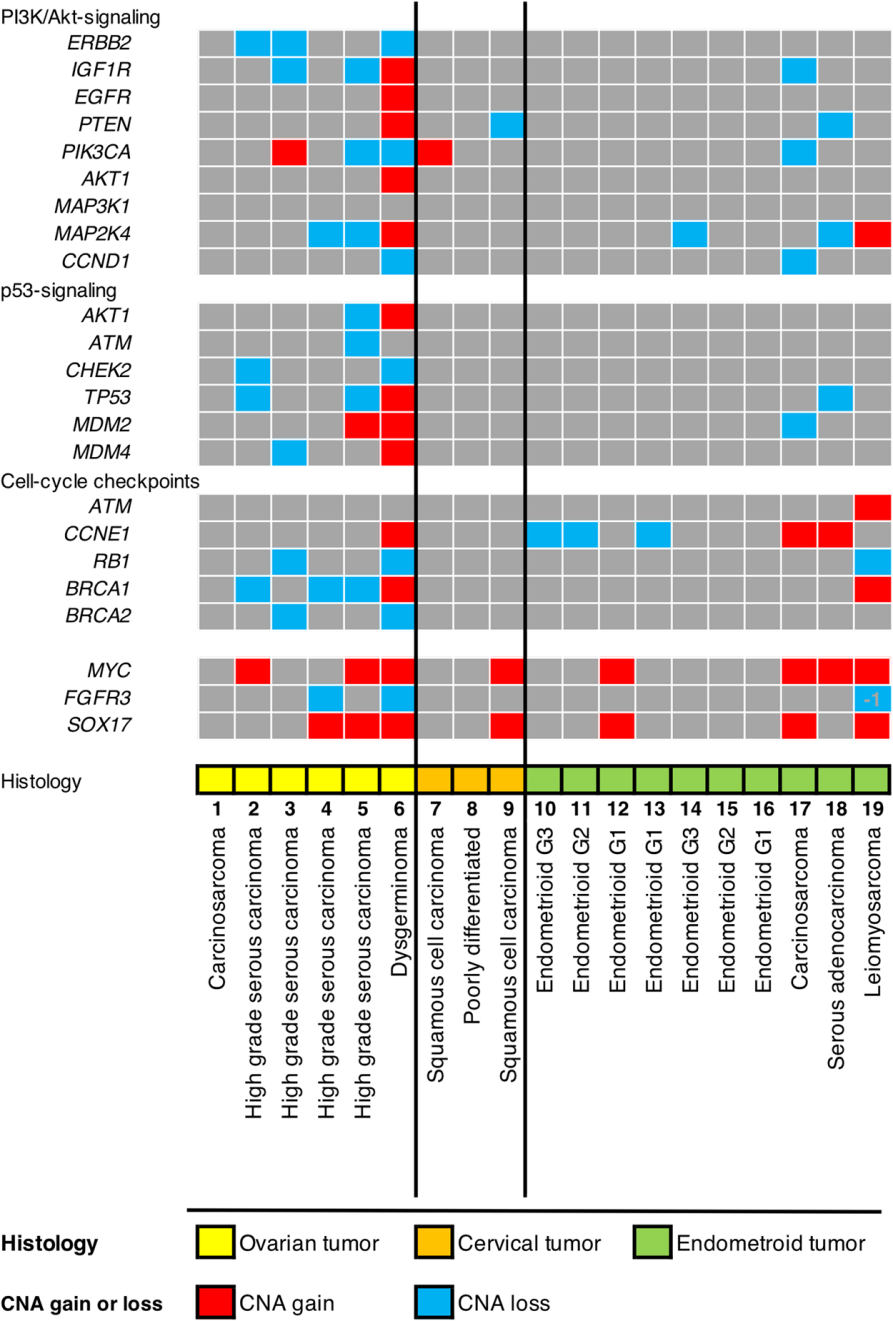
Each column corresponds to one cancer patient. Rows correspond to gain or loss of CNA in the sample. Sample histologies are shown in different colours representing ovarian tumour (yellow), cervical tumour (orange), and endometrial tumour (green) histologies. Gain and loss of CNA are shown as red and blue boxes, respectively.

Except for one carcinosarcoma case, all patients with CNA present in ovarian cancer expressed copy number loss of either *BRCA1* or *BRCA2*. The presence of CNA with high-grade serous carcinoma showed alterations of several genes involved in PI3K/Akt signalling. A patient with dysgerminoma also showed many alterations, even in stage I (Table 2, Fig. 2). In this study, only patients with early-stage cervical cancer were included, and amplification of *PIK3CA* was detected in one patient (Table 2, Fig. 2). In endometrial cancer, patients with carcinosarcoma, serous adenocarcinoma and leiomyosarcoma showed many alterations of cancer driver genes, such as *MYC* and *CCNE1* (Table 2, Fig. 2). In all patients with endometrioid endometrial cancer, CNA of genes related to p53-signaling was not detected. Only one patient with endometrioid endometrial cancer had CNA of *MYC*, and two had CNA of *ARID1A*.

**Table 2:** Detected copy number alterations in 6 ovarian cancer cases, 3 cervix cancer cases and 10 endometrial cancer cases mapped to reported gains and losses in the Genetech NIPT.

Sample number	Age (years)	Cancer type	Histology	FIGO stage	CNA size (Mb)	Detected CNA > 10 Mb				
1	77	Ovarian cancer	Carcinosarcoma	IIIB	31	19p13.3-19p13.11 gain				
2	46	Ovarian cancer	High- grade serous carcinoma	IIIB	565	1p36.33-1p34.3 gain	1q24.2-1q31.1 gain	5q32-5q35.3 gain	6p25.3-6p22.3 loss	8q22.1-8q24.3 gain
						8p23.3-8p11.21 gain	11p15.5-11p13 loss	12q14.3-12q21.32 gain	16p13.3-16q24.3 loss	17p13.2-17q21.33 loss
						19q13.2-19q13.43 loss	21q11.2-21q22.3 loss	22q11.1-22q13.33 loss	Xq24-Xq28 gain	
3	45	Ovarian cancer	High- grade serous carcinoma	IIIB	771	1q31.3-1q44 loss	1p31.1-1p21.1 loss	2q24.3-2q35 gain	3q26.1-3q28 gain	3q21.2-3q24 loss
						5q13.2-5q35.3 loss	5p15.33-5p15.31 gain	6q14.1-6q24.3 loss	6p25.1-6p22.3 gain	8q12.3-8q23.3 gain
						10q23.1-10q26.11 loss	10q26.2-10q26.3 gain	16q21-16q23.2 loss	17p13.3-17q21.32 loss	14q21.3-14q24.3 loss
						15q11.2-15q26.3 loss	16q21-16q23.2	17p13.3-17q21.32	18q22.2-18q23 loss	19q13.11-19q13.43 loss
4	45	Ovarian cancer	High- grade serous carcinoma	IIIC	981	1q25.3-1q41 gain	1p34.1-1p32.3 gain	2p23.2-2p16.1 gain	4p14-4q31.1 loss	5q13.3-5q15 loss
						6q25.2-6q27 loss	6p25.3-6p24.3 gain	7q32.3-7q35 gain	7p22.2-7p21.1 loss	9q21.11-9q22.31 loss
						10q25.1-10q26.2 loss	11p15.4 loss	11q22.3-11q23.3 gain	12q24.22-12q24.32 gain	12q23.3-12q24.21 gain
						13q21.1-13q34 gain	14q12-14q21.1 gain	15q21.1-15q23 loss	17p12-17q11.2 loss	17q21.2-17q23.3 gain
						18q21.2-18q22.1 loss	19p13.3-19p13.11 loss	20q11.22-20q13.2 gain		
5	49	Ovarian cancer	High- grade adenocarcinoma	IIIB	1767	1q22-1q32.1 gain	1q42.13-1q44 gain	2p25.3-2q37.3 loss	3p26.3-3q29 loss	7p12.3-7q32.3 gain
						8p11.21-8q24.3 gain	10q22.2-10q26.3 gain	11p15.5-11q25 loss	12p12.1-12q24.33 gain	13q12.11-13q34 loss
						14q11.2-14q32.33 loss	15q11.2-15q26.3 loss	16p13.3-16q24.3 loss	17p13.3-17q25.3 loss	18p11.32-18q22.3 gain
						20p13-20q13.33 loss	21q11.2-21q22.3 loss	22q12.3-22q13.33 loss	Xp22.33-Xq28 loss	
6	23	Ovarian cancer	Dysgerminoma	IA	2448	1q21.1-1q44 gain	1p34.2-1p11.2 loss	2p25.3-2q36.3 gain	3p26.3-3q29 loss	4p16.3-4q35.2 loss
						5q14.3-5q35.1 loss	4p16.3-4q35.2 loss	5q14.3-5q35.1 loss	8p23.3-8q24.3 gain	9q21.11-9q33.2 loss
						9p24.3-9q21.11 gain	10p15.3-10q26.3 gain	11p15.5-11q25 loss	12p13.33-12q24.33 gain	13q12.11-13q14.3 loss
						14q11.2-14q32.33 gain	15q11.2-15q26.3 gain	16p13.3-16q24.3 loss	17p13.3-17q25.3 gain	18p11.32-18q23 loss
						19p13.3-19q13.43 gain	20p13-20q13.33 gain	21q21.3-21q22.2 gain	22q11.1-22q13.33 loss	
7	51	Cervix cancer	Squamous cell carcinoma	IIB	21	3q26.32-3q29 gain				
8	48	Cervix cancer	Poorly differentiated adenocarcinoma	IB	89	16p13.3-16q24.3 loss				
9	43	Cervix cancer	Squamous cell carcinoma	IB	1077	1p11.2-1q44 gain	3p26.3-3q11.1 loss	3q11.1-3q29 gain	6p25.3-6p11.2 gain	6p11.2-6q27 loss
						7p22.3-7q36.3 loss	8p11.1-8q24.3 gain	8p23.3-8p11.1 loss	9p24.3-9q34.3 gain	10p15.3-10q26.3 loss
10	81	Endometrial cancer	Endometrioid G3	IIIA	62	19p13.3-19q13.43 loss				
11	77	Endometrial cancer	Endometrioid G2	IIIC	79	19p13.3-19q13.43 loss				
12	53	Endometrial cancer	Endometrioid G1	IA	93	8q11.1-8q24.3 gain				
13	69	Endometrial cancer	Endometrioid G1	IA	97	6q25.3-6q27 gain	19p13.3-19q13.43 loss	20q11.22-20q13.13 loss		
14	65	Endometrial cancer	Endometrioid G3	IIIC	102	16p13.3-16q24.3 loss	17p12-17q11.1 gain			
15	62	Endometrial cancer	Endometrioid G2	IA	248	1p36.33-1q44 gain				
16	61	Endometrial cancer	Endometrioid G1	II	248	1p36.33-1q44 gain				
17	25	Endometrial cancer	Carcinosarcoma	IIIA	821	3q26.1-3q26.32 gain	6q25.3-6q27 loss	8p12-8q24.3 gain	9p24.3-9q34.3 loss	10p15.3-10p11.22 gain
						11p15.5-11q25 loss	12p13.33-12q24.33 loss	15q11.2-15q26.3 loss	16p13.3-16q24.3 loss	19p12-19q13.43 gain
						22q11.1-22q13.33 loss				
18	72	Endometrial cancer	Serous adenocarcinoma	IV	834	1p35.1-1p13.1 loss	2p24.3-2p16.1 gain	3q26.32-3q27.2 gain	3q26.2-3q26.31 gain	4q13.1-4q28.3 loss
						5q12.3-5q35.3 gain	6p12.3-6q27 loss	7q22.1-7q36.3 loss	8q22.2-8q24.13 gain	8p22-8p12 loss
						9p24.3-9p21.1 loss	10p12.33-10p11.22 loss	10q21.2-10q22.1 loss	11p13-11p11.2 gain	11q14.1-11q14.3 gain
						13q21.2-13q34 gain	16q21-16q23.1 loss	17p13.3-17p11.2 loss	18q21.1-18q23 loss	18q11.1-18q12.3 gain
						19p13.3-19p13.2 loss	19q11-19q13.2 gain	20q13.32-20q13.33 gain	20q11.21gain	21q11.2-21q21.2 loss
						21q22.3 gain				
19	75	Endometrial cancer	Leiomyosarcoma	IB	1491	1p36.12-1q21.3 gain	2p25.3-2p14 loss	3p24.3-3q25.2 gain	4p14-4q35.2 gain	4p16.3-4p15.32 loss
						5p15.31-5q11.2 gain	6p25.3-6p22.3 loss	6q25.2-6q27 loss	7q11.21-7q35 loss	7p22.3-7p12.1 gain
						8p12-8q24.3 gain	9p24.3-9q21.11 loss	10q22.3-10q26.3 loss	11p15.5-11q25 loss	12p13.2-12p11.1 loss
						12p13.33-12p13.31 loss	13q13.1-13q14.2 loss	13q14.3-13q34 gain	14q11.2-14q21.1 gain	16q21-16q23.3 loss
						16p11.1-16q21 loss	17p13.3-17q25.3 gain	Xp22.32-Xq13.2 gain

### Comparison of PFS and OS with or without CNA

**Figure 3:**
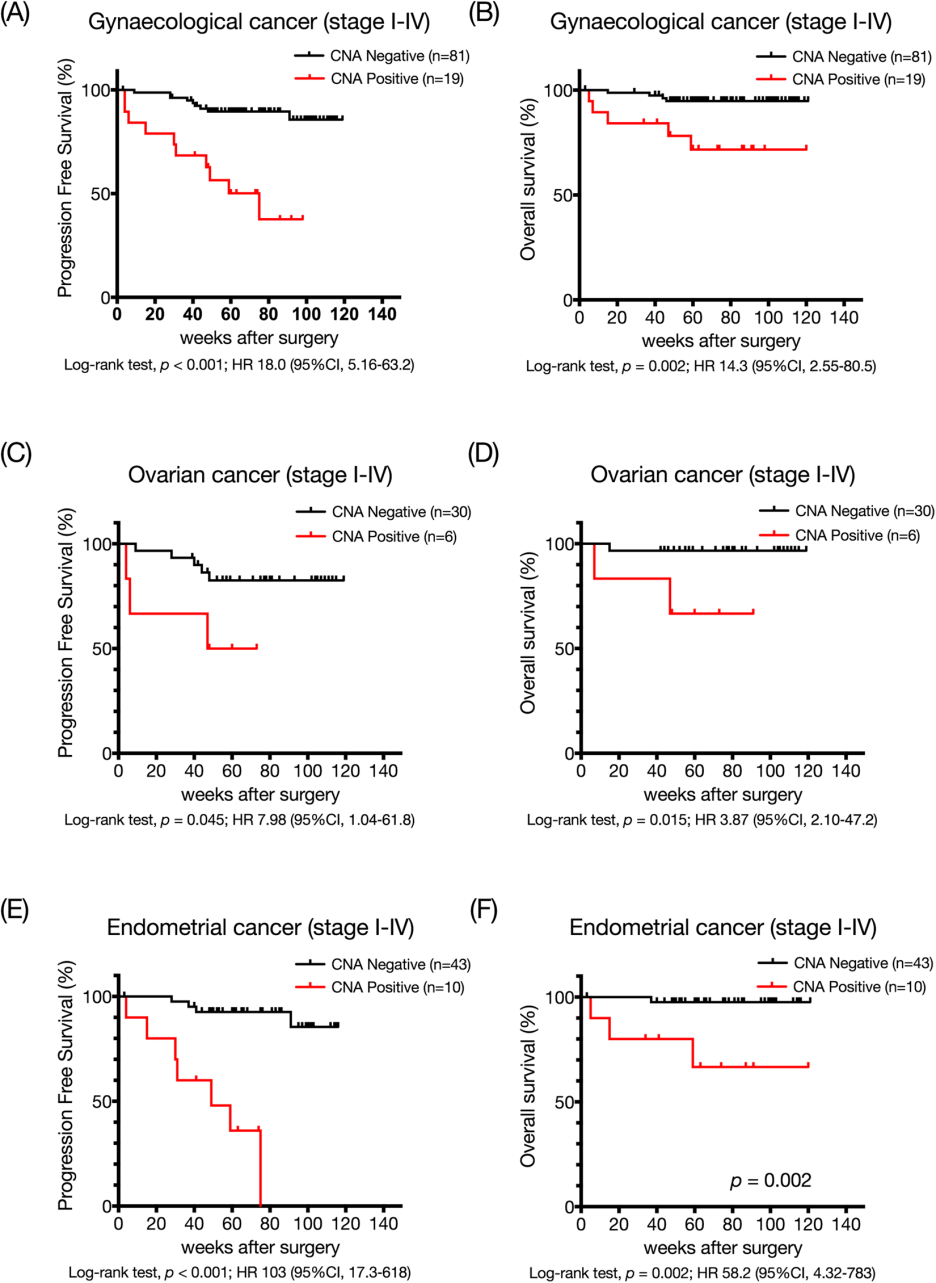
All stages of gynaecological cancer (**A**,**B**), ovarian cancer (**C**,**D**) and endometrial cancer (**E**,**F**). Red curves are for CNAs positive patients while black curves are for CNAs negative patients. *P*-values were calculated by log-rank tests for the differences in PFS and OS between CNAs positive and negative groups.

We examined whether preoperative CNA analyses may be associated with disease recurrence and survival after surgical resection regarding overall survival and progression-free survival looking at CNA positive and CNA negative in all participants and each histotype. In all patients with stage I–IV gynaecological cancer, patients with CNA had a shorter PFS and OS compared with those of patients without CNA (Fig. 3A and B). Kaplan–Meier analyses performed for the cases with stage I–IV ovarian cancer showed shorter PFS and OS (Fig. 3C and D). Similar trends were seen when the cases with stage I-IV endometrial cancer were analysed (Fig. 3E and F).

**Figure 4:**
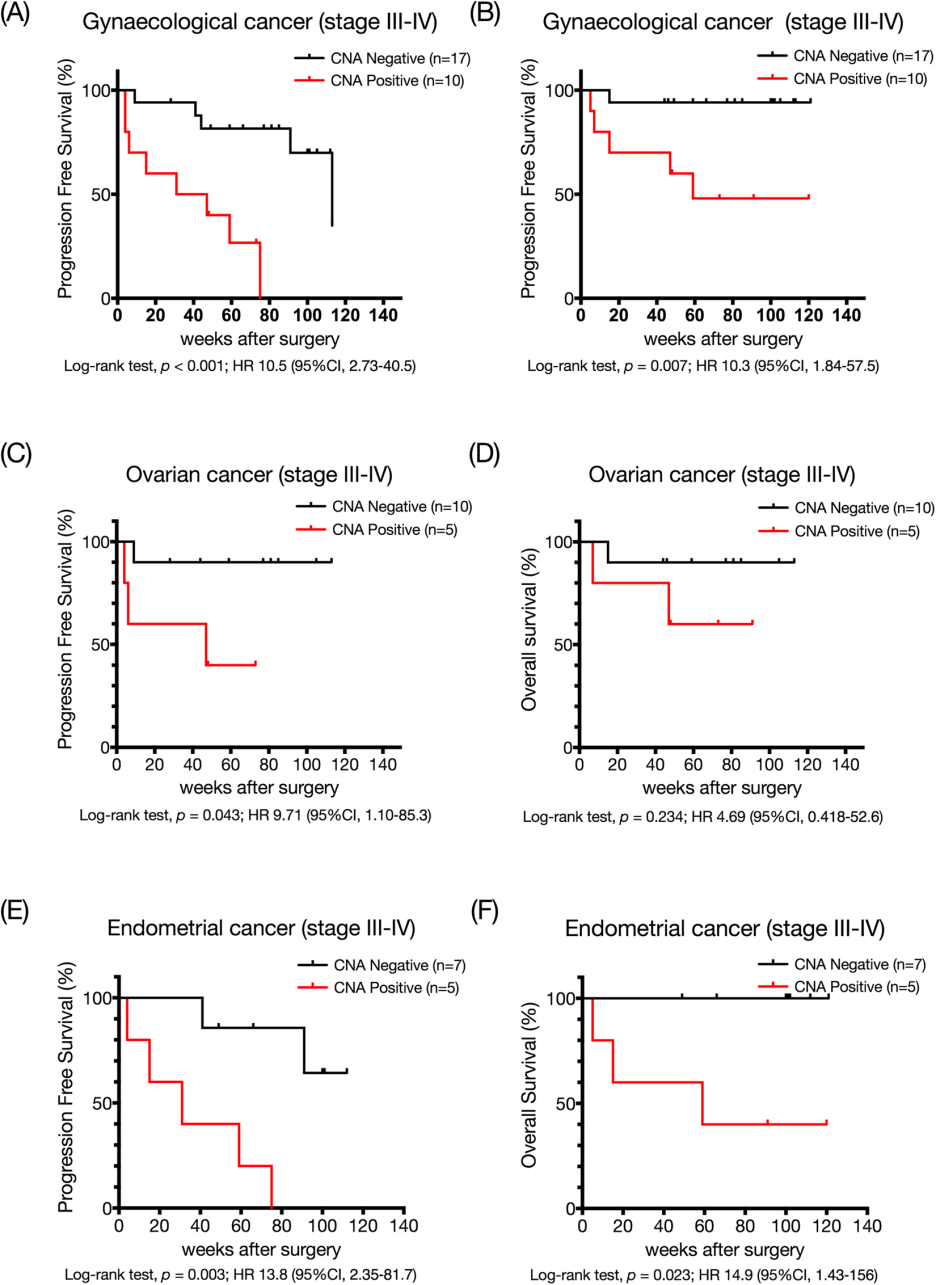
Advanced stages of gynaecological cancer (**A**,**B**), ovarian cancer (**C**,**D**) and endometrial cancer (**E**,**F**). Red curves are for CNAs positive patients while black curves are for CNAs negative patients. *P*-values were calculated by log-rank tests for the differences in PFS and OSl between CNAs positive and negative groups.

Next, we hypothesised that the detection of CNA was more likely to be associated with prognosis especially in advanced stage tumours. In all patients with advanced-stage gynaecological cancer, patients with CNA also had a shorter PFS and OS compared with those of patients without CNA (Fig. 4A and B). In the cases with stage advanced-stage ovarian cancer, Kaplan–Meier analyses showed shorter PFS but didn’t find the difference of OS (Fig. 4C,D). The same stratification revealed an association between the presence of CNA in advanced-stage endometrial cancer patients with shorter PFS and OS (Fig. 4E and F).

## Discussion

The present study has revealed that an NIPT platform with low-coverage whole-genome sequencing and MPS for detecting CNA in plasma could identify not only a surprisingly high burden neoplasm but also early stage gynaecological cancers and predict their recurrence, particularly in advanced stages. As such, we believe these molecular findings offer a future promise of predicting cancer recurrence in women with gynaecological cancer after initial surgery as well as a rare opportunity to explore the processes of defining gynaecological cancer in patients with the non-invasive prenatal diagnosis.

The present study used the SeqFF method^28^ to detect CNAs in plasma, a cell-free DNA count-based approach that was developed to enable a direct estimate of foetal DNA fraction from routine NIPT data without any additional requirements. This method is based on the findings from maternal serum with regards to fragment length differences between foetal and maternal cell-free DNA^29^. Here, the SeqFF method was used to detect CNA in plasma based on the hypothesis that the fragment length of ctDNA differs from cell-free DNA. This theory is supported by reports that utilised amplicons of varying length to identify sizeable categorical size differences between ctDNA are associated with cancer and cell-free DNA from healthy controls^30–32^. In the present study, CNA detection was used by whole-genome sequencing (WGS). CNA detection by high throughput sequencing still faces analytical challenges due to the rampant biases and artefacts that are introduced during library preparation and sequencing. Studies are gradually producing more robust detection of CNAs, particularly in targeted sequencing panels using hybridisation capture approaches. Targeted sequencing panels focus on individual genes or specific regions of interest. This method supports the detection of identified variants within targeted regions and, but it needs previous knowledge of related regions of the genome, and the variability in efficiency of amplification during library preparation leads to jagged amplicon coverage from one experiment to another. Although several targeted sequencing methods also allow the detection of CNAs, WGS presents an additional advantage of unbiased sequencing and cost^33^.

In general, increased detection rates can be achieved with a higher input cell-free DNA and higher cancer stages^34^. Numerous patient cohorts have enabled the precise characterisation of CNAs that predict clinical outcomes in high-grade serous ovarian cancer^35,36^. Moreover, in cell model study, CNAsdiffered between matched highly and minimally invasive/migratory subclones of ovarian cancer^37^. Also, in endometrial cancer, copy number high in a tumour have shown the poorest disease-free survival^27^. In other cancers, low‐pass whole‐genome sequencing and evaluated CNA in cell-free DNA demonstrates progressive CNA accumulation from stage I to IV and significant association of specific genomic loci with overall survival or metasis^38,39^. We have shown that CNAs, as a global measure of the level of CNA across cell-free DNA in plasma, is associated with progression-free survival and overall survival of gynaecological cancer and moreover especially in the advanced stage of ovarian cancer and endometrial cancer. This study further confirmed potential clinical applications of cell-free DNA based CNAs as a promising biomarker for cancer prognosis, especially for advanced stage cancers.

Genomic alterations such as CNAs are known to harbour drivers of carcinogenesis. Several known CNA drivers in cancers include receptor tyrosine kinases, which are targets for drug therapies^40^. Trastuzumab, an antibody to ERBB2 used in breast cancer therapy, provides an excellent example of an amplified cancer gene as a specific therapeutic target^41^. Some high-level amplifications have been highlighted as predictive biomarkers, including *CCNE1*, *RB1*, *MYC*, *ERBB2*, *PIK3CA*, *EVI1*, *AKT2*, *NOTCH3* and *FGFR1* in ovarian cancer^35,42^. Frequent increases in DNA copy number at chromosomal region 8q24.3, which contains cancer-related genes such as *PTP4A3*, have been reported to serve as a prognostic marker in ovarian carcinomas^43^. This region is also frequently amplified in endometrial cancer^44^. Another study and analysis of the cancer genome atlas data suggested that chromosomal gains in endometrial endometrioid adenocarcinoma were observed with relatively high frequencies in 1p36–p31 and 1q12–q44^45^. Our data reveal that three cases of endometrial endometrioid adenocarcinoma had amplification of 1p36–p31 and 1q12–q44 or 8q24.3. Carcinosarcoma, serous adenocarcinoma and leiomyosarcoma cases expressed CNA in many regions.

There are several limitations of this study. First, plasma sequencing data was not compared to the tumour DNA and this made it difficult to confirm the tumour origins of the CNA. Second, we only used commercially based techniques for our calculations and did not evaluate the association between the amount of cell-free DNA and size distribution. The principle that tumour DNA is detectable in plasma using NIPT sequencing platforms has been previously reported^1–5^. Third, the sample numbers were too small to perform analyses stratified by histological subtypes within the tumour site groups, which could be confounding the survival analysis. Further studies preferably using prospective CNA samples are required for clinical validation of the method and confirmation of the origin of the CNA. The costs and feasibility of sequencing and CNA analysis are continually decreasing. However, the costs are still high to use the genomic data in a clinic. Our data showed the commercial method for non-invasive CNA analysis could apply for using publicly available genomic data to provide the information of the recurrence of cancer in gynaecological cancer patients before surgery.

In summary, we have demonstrated that CNA in patients with gynaecological cancer detected using a commercially based NIPT platform could predict recurrence after initial surgery, particularly in advanced stages. The ability of the NIPT platform to identify CNA in plasma has numerous potential clinical applications and provides the opportunity to detect potentially aggressive cancers in pregnant women. As sequencing techniques develop and become more affordable, non-invasive and longitudinal surveillance may become a valuable tool available to clinical oncologists. We focused on the application of CNA in plasma and believed that this method has a broader scope for genetic diagnoses, such as the analysis of ctDNA to detect cancer and predict prognosis, although its clinical utility must be further studied. The software could be used to perform one or more steps in the processes and, regardless of aetiology, detection of ctDNA augmented for cell-free DNA fragment lengths may lead to non-invasive diagnosis of malignancy, improved detection of tumour recurrence, and better monitoring of response to therapy.

## Methods

### Patient cohort and sample collection

**Figure 5:**
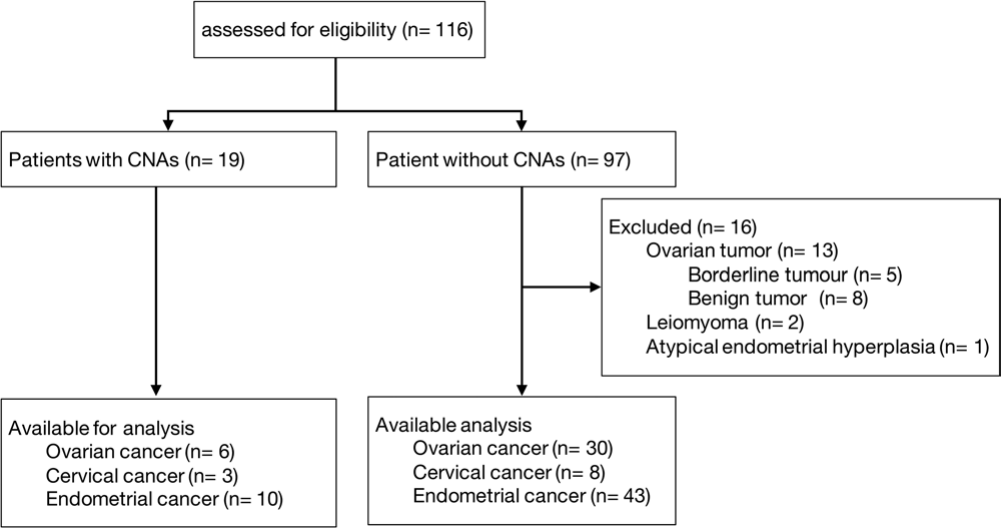
A flowchart illustrating the progress of subjects through the prospective study. 16 participants in the patients without CNAs were excluded because they were diagnosed with not cancer. CNA, copy number alterations in cell-free DNA.

We performed a case-control study of patients with gynaecological cancer recruited from Showa University Hospital. All clinical investigations were conducted according to the principles expressed in the Declaration of Helsinki. The study was approved by the ethical committee of the Show University Hospital (approval no. 229). Written informed consent about this study was obtained from all patients before undergoing surgery at our institution. We enrolled 116 participants including 100 gynaecological cancer patients between January 2016 and June 2017 (Fig. 5). Blood samples were collected taken within a week before surgery. Final diagnoses of gynaecological cancer were analysed using histopathology. The staging was performed at baseline for all patients using computed tomography scan. After surgery, patients were treated with adjuvant hormone therapy, radiotherapy or chemotherapy as per the standard local practice.

### Blood processing and DNA extraction

A sample of 10 mL of whole blood was collected in Cell-Free DNA BCT tubes (BCT tubes; Streck, Omaha, NE), centrifuged at 1600 × *g* for 15 min at 25 °C, and the plasma fraction was collected and centrifuged for a second time at 2500 × *g* for 10 min at 25 °C. After the second spin, the plasma was transferred into barcoded tubes and immediately stored at ≤70 °C until DNA was extracted. Cell-free DNA was extracted from 4 mL of patient plasma using the QIAamp Circulating Nucleic Acid Kit (Qiagen, Gaithersburg, MD, USA).

### Library preparation, quality control and sequencing

A volume of 40 mL of extracted cell-free DNA was used to prepare libraries using the NEBNext Ultra DNA Library Prep Kit for Illumina (New England Biolabs, Inc.; Ipswich, MA, USA), using custom index adapters modified to create 96 unique molecular barcodes with a minimum of three base edit distance^46^. Automated library preparation was performed on the Zephyr liquid handler (Perkin Elmer; Waltham, MA) using 96 unique index barcodes, enabling up to 96 sample multiplexing. Libraries were quantified on the LabChip GX (Perkin Elmer; Waltham, MA). All libraries were normalised to 1.6 nM, multiplexed and sequenced on HiSeq. 2500 with 27 sequencing cycles of the cell-free DNA insert and an additional eight sequencing cycles for the index barcodes.

### Sequencing analysis

Sequencing reads were aligned to the human reference genome (hg19) using Bowtie 2^47^. Reads mapped to each chromosome were aggregated using nonoverlapping 50 kbp-genomic segments. Regions were excluded from analysis based upon high variance, low capability, or a high percentage of repetitive elements as described by Jensen *et al*.^48^. Sequencing reads corresponding to the remaining 50 kbp-genomic segments were adjusted for sequencing biases as described in detail by Zhao *et al*.^49^. Briefly, sequence reads were first processed with a LOESS-based, sample-specific adjustment, and a principal component analysis-based smoothing was utilised to remove higher order artefacts with a population-based correction.

### Analytical methods and genome-wide detection of abnormalities

A multivariate model was derived to predict the tumour fraction from the regional autosome read depth coverage from single-end sequencing to calculate tumour DNA fraction. The amount of CNV can also be used to estimate the tumour DNA fraction, and bins located on chromosomes 13, 18, 21, X and Y are excluded from this method. To determine the association between the response and predictor variables, i.e. the model coefficients, various statistical modelling methods can be employed. Detailed bioinformatics analysis of the previously sequenced DNA sample was performed, and mapped sections of the human genome were analysed using circular binary segmentation (CBS) to identify CNA. The measured Z-scores form part of an enhanced version of Chromosomal Aberration DEcision Tree (CADET) previously described in detail by Zhao *et al*.^49^. CADET incorporates z-statistics for a CBS-detected CNA to assess the statistical significance and a log odds ratio to provide a measure of the likelihood of an event being real, based on an observed fraction of DNA across the genome^49^. To further improve the specificity of CNA detection, bootstrap analysis was performed as an additional measure of the confidence of the candidate CNA. The within-sample read count was compared with a standard population and quantified by bootstrap confidence level (BCL). To assess within-sample variability, bootstrap resampling (described below) was applied to every candidate CNA^50^. For each identified segment within the CNA, the median shift of segment fraction from the average level across the chromosome was calculated. This median change was then corrected to create a read count baseline for bootstrapping. Next, a bootstrapped segment of the same segment length as the candidate CNA was randomly sampled with replacement from the baseline read counts. The median shift was then applied to this bootstrapped fragment and calculated as follows:

$$Segment\,fraction=\frac{{\rm{\Sigma }}\,{\rm{read}}\,{\rm{counts}}\,{\rm{within}}\,{\rm{segent}}}{{\rm{\Sigma }}\,read\,counts\,across\,the\,autosome}$$

This process was repeated 1000 times to generate a bootstrap distribution of segment fractions for an affected population. The median chromosome fraction was calculated specific to each flow cell while the median absolute deviation was a constant value derived from a static median absolute deviation. A threshold was then calculated as the segment fraction that was at least 3.95 median absolute deviations away from the median segment fraction of the reference distribution. Lastly, the BCL was calculated as the proportion of bootstrap segments whose fractions had absolute z-statistics above the significance threshold^50^. A chromosome was classified as having an amplification or a deletion if:

$$|{Z}_{CBS}|\ge 3.95,\,LO{R}_{CBS} > 0,\,BCL\,\ge 0.99,\,and\,|{Z}_{CBS}| < \alpha |{Z}_{CHR}|$$

The comparison $|{Z}_{CBS}| < \alpha |{Z}_{CHR}|$ was used to distinguish a whole chromosome event from a subchromosomal event and denoted a type 1 error for misclassification of abnormalities as aneuploidy. Simulations showed that α = 0.8 resulted in a misclassification of foetal abnormalities at close to 0%^50^; therefore, this value was used in the present study. Using this scale, we counted identified CNA as gains or losses if they exceeded 10 Mb from the expected diploid coverage. These parameters were only used for visual interpretation of the data and were not intended to identify cancer signatures.

### Statistical analysis

Various methods were used to determine significance. Differences in means of unpaired samples were tested using Mann–Whitney *U* test (such as for comparisons involving the SeqFF value in plasma among the cancer population). We compared progression-free survival (PFS) and overall survival (OS) between patients with CNA present in plasma using the log-rank test for univariate analyses and the Cox proportional hazards for multivariate analyses. Computer analyses were performed using Prism 7 (Graphic Pad Software Inc.). Statistical significance was defined as p < 0.05.

## Electronic supplementary material

Dataset 1
