# Publisher Correction: Massively parallel sequencing of cell-free DNA in plasma for detecting gynaecological tumour-associated copy number alteration

**DOI:** 10.1038/s41598-018-34168-2

**Published:** 2018-10-23

**Authors:** Makoto Nakabayashi, Akihiro Kawashima, Rika Yasuhara, Yosuke Hayakawa, Shingo Miyamoto, Chiaki Iizuka, Akihiko Sekizawa

**Affiliations:** 1 Department of Obstetrics and Gynecology, Showa University School of Medicine, 1-5-8 Hatanodai, Shinagawa-ku, Tokyo 142-8666 Japan; 2 Division of Pathology, Department of Oral Diagnostic Sciences, Showa University School of Dentistry, 1-5-8 Hatanodai, Shinagawa, Tokyo 142-8666 Japan; 3 Information System Department GeneTech, Inc. 2-6-7 Kazusa-Kamatari, Kisarazu, Chiba 292-0818 Japan

Correction to: *Scientific Reports* 10.1038/s41598-018-29381-y, published online 25 July 2018

In the original version of this Article, Makoto Nakabayashi was incorrectly listed as corresponding author. The correct corresponding author for this Article is Akihiro Kawashima. Correspondence and request for materials should be addressed to kurobei343@med.showa-u.ac.jp. This error has now been corrected in the HTML and PDF versions of the Article.

